# An Unusual Presentation of Diffuse Large B-Cell Lymphoma With Penile Metastasis

**DOI:** 10.7759/cureus.65222

**Published:** 2024-07-23

**Authors:** Prashanth Reddy Yella, Prachi P Jagani, Vishva Patel, Ravi P Jagani, Vaiishnavi Ramesh, Priya Elsa Skaria, Abhinav Chandra

**Affiliations:** 1 Internal Medicine/Hospital Medicine, Yuma Regional Medical Center, Yuma, USA; 2 Pre-Medical Sciences, Richmond Gabriel University, Kingstown, VCT; 3 Medical Sciences, Gujarat Medical Education and Research Society (GMERS) Medical College, Ahmedabad, IND; 4 Family Medicine, Yuma Regional Medical Center, Yuma, USA; 5 Hematopathology, Yuma Regional Medical Center, Yuma, USA; 6 Oncology, Yuma Regional Medical Center, Yuma, USA

**Keywords:** pet ct scan, metastatic diffuse large b-cell lymphoma, r-chop chemotherapy, penile mass, extranodal diffuse large b-cell lymphoma

## Abstract

Diffuse large B-cell lymphoma (DLBCL) is a high-grade B-cell lymphoma that most commonly presents with lymph node involvement. Extranodal manifestations are seen in around 40% of the cases involving the gastrointestinal tract, thyroid, testes, brain, and breast, among many others. However, penile metastasis is extremely rare and often overlooked in routine clinical evaluations. We present the unique case of a 79-year-old man with a history of DLBCL with extranodal involvement who achieved remission after completing five cycles of chemotherapy and presented eight months later with a new penile mass. A PET-CT scan of the skull to mid-thigh revealed bilateral pulmonary nodules, multiple lesions in the pancreas, retroperitoneal nodules, and an increased uptake at the base of the penis, leading to a biopsy of the penile mass that confirmed recurrent DLBCL with penile metastasis. The patient subsequently underwent surgical excision of the lesion and additional chemotherapy. This case underscores the importance of considering atypical sites of involvement in DLBCL patients and emphasizes the need for a timely diagnostic workup to ensure early detection and accurate diagnosis. By raising awareness of this rare manifestation and promoting comprehensive evaluations, we can potentially improve patient outcomes and facilitate the development of more effective treatment strategies.

## Introduction

Diffuse large B-cell lymphoma (DLBCL) represents the most prevalent subtype of non-Hodgkin lymphoma (NHL), accounting for 30% of all B-cell NHL cases worldwide. Although it is more commonly diagnosed in males around the seventh decade of life, DLBCL can occur at any age [1]. It is a high-grade lymphoma with the usual clinical presentation involving widespread lymph node enlargement, yet approximately 40% of the cases exhibit extranodal manifestations. The most frequently affected extranodal sites are the gastrointestinal tract, including the stomach, small intestine and large intestine, testis, thyroid gland, skin, breast, bone, and central nervous system [2]. Involvement of the penis, however, is infrequent and is not commonly considered in routine clinical evaluations. It often signifies advanced disease [3].

In this case report, we present the case of penile metastasis in a 79-year-old man with a history of DLBCL. The patient had undergone five cycles of chemotherapy and was subsequently found to have an enlarging mass on the shaft of his penis. Imaging studies with a PET-CT scan demonstrated increased uptake at the base of the penis, with a subsequent biopsy that confirmed the recurrence of DLBCL. This report highlights the importance of considering unusual sites of involvement in patients with DLBCL. It aims to educate clinicians on the diagnostic workup necessary for early detection and initiation of treatment, thereby improving patient outcomes.

## Case presentation

We present the case of a 79-year-old man with a past medical history of atrial fibrillation, osteoarthritis, type 2 diabetes mellitus, and a history of smoking (seven pack-years) who presented to the ED after sustaining a fall while repairing a roof. In the ED, a CT scan of the chest, abdomen, and pelvis with contrast revealed a fracture of the left inferior pubic ramus and anterior acetabulum, along with lucency in the posterior right inferior pubic ramus extending into the acetabulum. These findings raised concerns about a benign or malignant neoplasm. Additionally, the patient had an atypical hemangioma versus a malignant lesion in the T9 thoracic vertebra and multiple spondyloses with narrowing of the neural foramina on CT of the thoracic and lumbar spine. Due to a lack of available specialists, the patient was transferred to a tertiary care center for further management.

The patient was also found to have significant iron deficiency anemia and was advised to undergo a colonoscopy for evaluation. The colonoscopy revealed a partially obstructing mass in the mid-ascending colon, suspicious of possible malignancy. Biopsies were obtained, and a tattoo was placed adjacent to the lesion. The biopsy results confirmed DLBCL of the germinal center subtype in the ascending colon. A PET-CT scan from the skull to the mid-thigh initially showed metabolic activity confined to the ascending colon area. The patient began R-CHOP chemotherapy and completed five cycles over five months. After the fifth cycle, he experienced poor tolerability, including severe diarrhea and pancytopenia, leading to the omission of the sixth cycle. At the end of treatment, a PET-CT scan showed complete remission.

**Figure 1:**
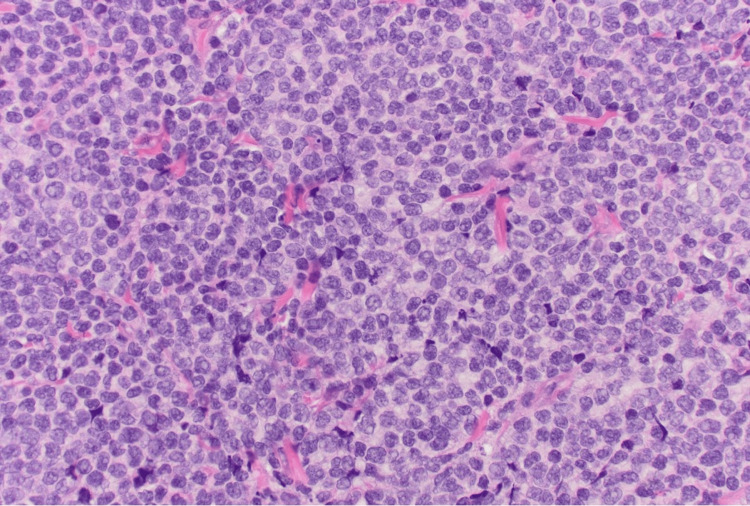
Diffuse sheets of pleomorphic large cells with irregular nuclear contours, vesicular chromatin, occasional prominent nucleoli, and frequent mitotic figures (H&E stain, 400× magnification)

**Figure 2:**
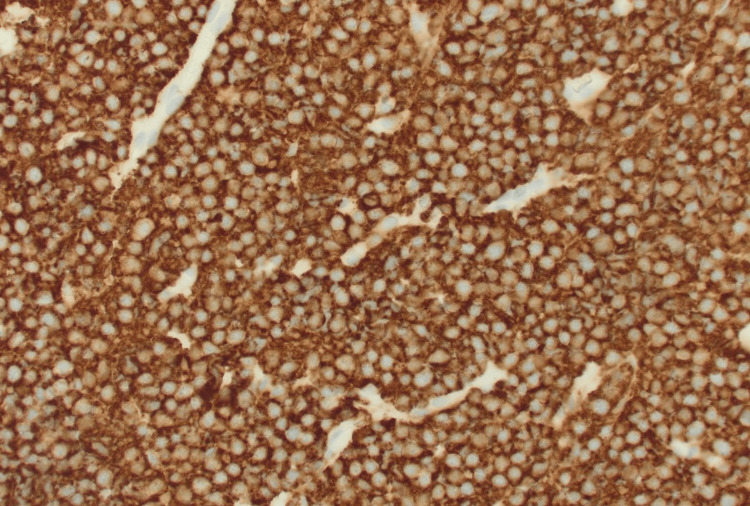
Diffuse strong membranous positivity of large cells confirms B-cell lineage (CD20 immunohistochemical stain, 400× magnification)

**Figure 3:**
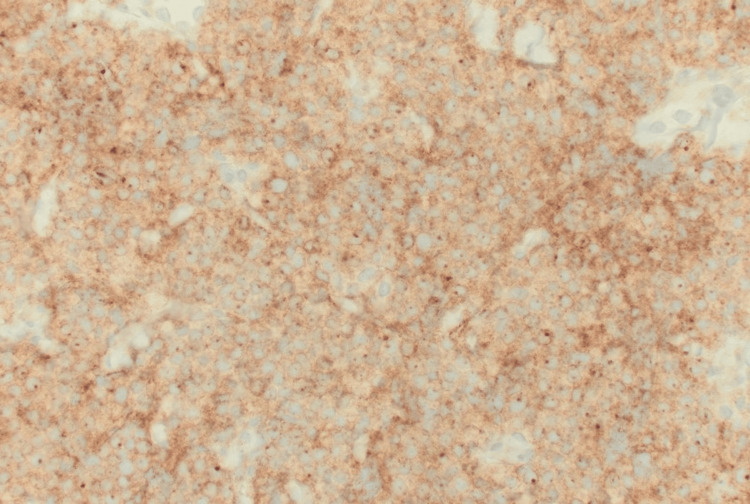
Large B-cells are positive for CD10, confirming germinal center origin per the Hans algorithm (CD10 immunohistochemical stain, 400× magnification)

**Figure 4:**
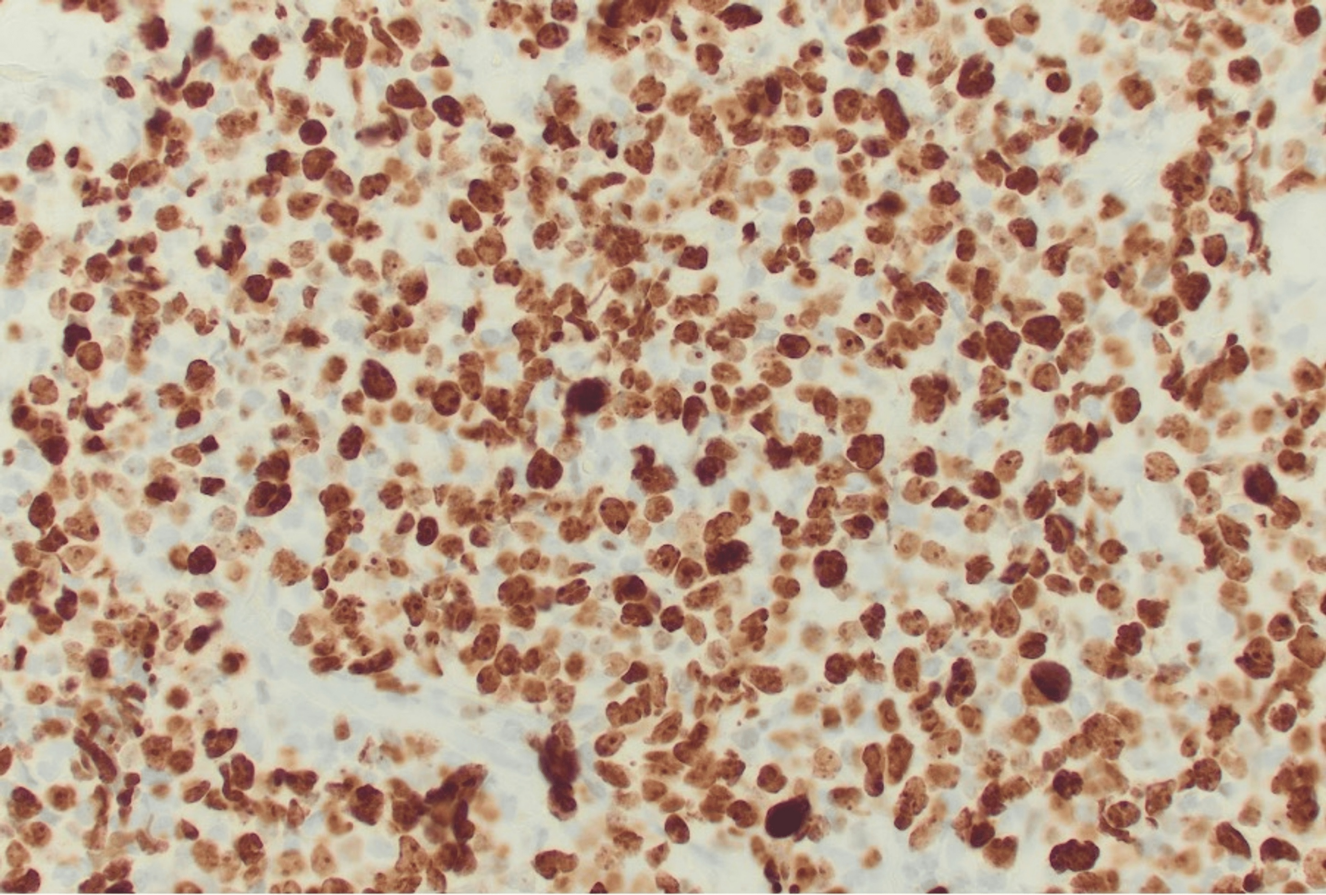
Ki67/MIB1 immunohistochemical stain demonstrates a proliferation index of approximately 90%, consistent with high-grade malignancy (400× magnification)

Approximately eight months after completing treatment, the patient presented to the oncologist with new white lesions on the penis. Physical examination revealed a normal-appearing male with a 2-3 cm mass on the shaft of the penis and no associated inguinal lymphadenopathy. A routine CT scan of the chest, abdomen, and pelvis showed new suspicious pulmonary and retroperitoneal nodules, as well as an enhancing mass along the right aspect of the penile shaft. A repeat PET-CT from the skull to the mid-thigh demonstrated increased and new focal metabolic activity around the pulmonary nodules, pancreas, left neck, right supraclavicular region, left pelvis, right inguinal region, and right nasopharynx, as well as around the base of the penis. This distribution was atypical for lymphoma, raising suspicion of a new neoplastic process. The patient was referred to a urologist for an incisional biopsy of the penile mass, which confirmed the recurrence of DLBCL of the germinal center subtype (histology images of the penile mass are shown in Figure 1, Figure 2, Figure 3, and Figure 4).

He was started on lenalidomide and rituximab chemotherapy with palliative intent due to the extensive spread of the lymphoma. Given his age, lack of social support, and poor tolerability, he was deemed a poor candidate for chimeric antigen receptor T cell therapy (CAR T cell therapy). The patient continued chemotherapy with lenalidomide and rituximab and completed four cycles, showing a favorable response to treatment. He was being closely followed by the oncologist.

## Discussion

DLBCL is the most common histologic subtype of NHL, accounting for 25-30% of all NHL cases [4]. Typically, it presents in men at the median age of 64, accounting for approximately 55% of cases [5]. The histology is characterized by centroblasts and immunoblasts, mature B-cells that grow larger than normal due to a failure to respond to typical size-regulating signals during development [4]. Many DLBCL cases can have extranodal involvement; however, penile involvement is sporadic and usually not examined in normal clinical examinations. Penile cancer is rare compared to DLBCL, representing less than 1% of all malignancies in the United States and Europe. Penile metastasis is even less frequently reported in the literature [6,7]. Chu et al. studied penile lymphomas and identified only 48 instances, of which DLBCL was the most frequent subtype with 14 cases [8]. This displays the rare nature of DLBCL with penile metastasis.

The typical presentation of DLBCL varies depending on how the patient initially presents and the extent of their nodal involvement [8]. Most commonly, it presents with general symptoms such as immunosuppression, thrombocytopenia, and anemia. In about 30% of patients, there are also systemic symptoms present, such as lethargy, generalized malaise, unintended rapid weight loss, nocturnal hyperhidrosis, and recurrent fever, all associated with B-cell proliferation [3]. In terms of nodal involvement, the staging system based on the Lugano classification is used to determine the extent and progression of the disease [9]. In stages I and II of the illness, there may be nodes present in the neck or abdomen and extranodal involvement among patients in stages III and IV of the illness [10]. The most common presentation specific to DLBCL with penile metastasis is the presence of a palpable mass or lump on the penis that may be painless or associated with discomfort, pain, or ulceration [11]. In some cases, patients may complain of frequent and painful urination [12].

Diagnosing penile metastasis from DLBCL can be challenging as it frequently presents with symptoms that mimic common conditions like penile carcinoma or infections. Therefore, a systematic approach is essential in such cases. The utilization of imaging techniques, including PET-CT and MRI, can provide detailed anatomical information, aiding in identifying both primary and metastatic lesions [12]. Specifically, PET-CT scans are particularly useful for staging DLBCL, assessing disease extent, locating metastatic sites, and guiding biopsy selection because they are able to detect and analyze metabolic activity. To confirm the diagnosis of a patient, histopathological examination of tissue samples from the penile mass obtained through biopsy is mandatory. The samples tend to be covered by stratified squamous epithelium with or without ulceration and demonstrate infiltration by diffuse sheets of neoplastic large B-cells [12]. In our presentation, after eight months of chemotherapy, when the patient returned complaining of white lesions around the penis and suspicious findings of a mass along the right aspect of the penile shaft on a CT scan, a repeat PET-CT scan was performed. The findings on the PET-CT showed increased metabolic activity around the base of the penis. Suspecting a new neoplastic process, biopsies were taken, which confirmed the diagnosis. The diagnostic approach of using a PET-CT scan to determine the presence of metabolic activity aligns with the guidelines for DLBCL surveillance and disease monitoring post-therapy.

Over the years, CAR T cell immunotherapy has demonstrated promising results in eradicating very advanced leukemias and lymphomas [13]. It involves customizing treatment for each patient by collecting T cells from them and modifying them to produce proteins on their surface called CARs. The CARs recognize and bind to specific proteins, or antigens, on the surface of cancer cells. This sort of treatment necessitates a robust social support system and a high tolerance for potential side effects due to the demanding treatment process [13]. Our patient lacked these prerequisites, rendering CAR T cell immunotherapy unsuitable. Instead, the standard treatment for DLBCL involves chemoimmunotherapy with four drugs known as cyclophosphamide, doxorubicin, vincristine, and prednisone (CHOP), plus the monoclonal antibody rituximab. For patients with early-stage DLBCL (stages I and II) involving only one or two lymph node groups, R-CHOP is given in three to six cycles. However, for advanced-stage DLBCL (stages III/IV), the recommended initial treatment is with six cycles of R-CHOP or rituximab, polatuzumab, vedotin, cyclophosphamide, doxorubicin, and prednisone (R-pola-CHP) [14]. In cases where DLBCL recurs or becomes resistant to initial treatment, high-dose chemotherapy followed by autologous stem cell transplantation may be used as a salvage therapy. In the case of DLBCL with penile metastasis, R-CHOP therapy is preferred as it has been shown to preserve penile functions [12]. Following the first PET-CT scan and confirmation of DLBCL recurrence on biopsy, the patient underwent five cycles of R-CHOP chemotherapy. In previous case series, the reported disease-free survival rates for DLBCL with penile metastasis ranged from six to 48 months [7]. Comparatively, our patient experienced complete remission, but after eight months, a significant relapse occurred. Due to past side effects like diarrhea and pancytopenia with R-CHOP therapy, he was switched to lenalidomide and rituximab chemotherapy with palliative intent to improve his quality of life. Alternative regimens to R-CHOP had not shown superior outcomes and were more toxic in previously reported cases [15]. However, the results in phase 2 of the REAL07 trial showed that lenalidomide with R-CHOP21 was effective and safe in elderly patients with untreated DLBCL [15].

The Ann Arbor classification, used to stage DLBCL, does not consistently predict long-term survival. To address this, the International Prognostic Index (IPI) was developed, incorporating clinical factors to predict outcomes. Modified versions like the International Index and Age-Adjusted International Index improve accuracy compared to Ann Arbor. Additionally, R-CHOP therapy has significantly improved DLBCL prognosis by increasing remission rates and overall survival. Due to DLBCL’s aggressive nature and tendency to present at later stages with distant metastases, the prognosis for patients with penile metastasis is typically guarded [16]. The prognosis for patients depends on factors like overall health, treatment response, and the extent of metastasis. In this case, despite achieving complete remission initially with R-CHOP chemotherapy, the patient’s age (79 years) and significant comorbidities (atrial fibrillation, osteoarthritis, and type 2 diabetes mellitus), along with poor chemotherapy tolerance, suggest a less favorable outlook. Transitioning to lenalidomide and rituximab chemotherapy with palliative intent aims to manage symptoms and improve quality of life. Despite advancements in treatment, therapeutic challenges persist, particularly for the elderly with comorbidities. While some individuals may respond well with appropriate care and close monitoring, there are currently no other curative options available for this complex condition [8].

## Conclusions

DLBCL with penile metastasis is a rare disease recurrence pattern, involving an extranodal site. Given its rarity, atypical presentation, and multidisciplinary nature, maintaining vigilance is crucial when considering this manifestation. The diagnosis heavily relies on imaging studies, which play a pivotal role in guiding and confirming the diagnosis. Cytogenetic and molecular genetic studies are not required; however, they may support solidifying the diagnosis. Due to the poor prognosis and extreme aggressiveness of the disease, early detection is vital. Aiming to improve patient survival rates, the treatment is individualized and typically involves systemic chemotherapy. This case study hopes to educate clinicians on diagnostic criteria and imaging modalities, with the ultimate goal of improving patient outcomes and finding a cure, as none currently exists.
